# Fungus That Struck the Brain: A Case of Invasive Sino-Orbital Aspergillosis Causing a Stroke

**DOI:** 10.7759/cureus.84327

**Published:** 2025-05-18

**Authors:** Nidha Shapoo, Jumana Chalabi, Paul Huang, Seth Lieberman, Noella Boma

**Affiliations:** 1 Internal Medicine, New York Medical College, Metropolitan Hospital Center, New York, USA; 2 Neurosurgery, New York Medical College/Bellevue Hospital, New York, USA; 3 Otolaryngology, New York Medical College/Bellevue Hospital, New York, USA

**Keywords:** carotiditis, invasive aspergillosis, orbital apex syndrome, sino-orbital infection, stroke

## Abstract

Invasive sino-orbital aspergillosis is a rare but potentially fatal fungal infection that predominantly affects immunocompromised individuals. It can lead to severe complications such as internal carotid artery invasion and ischemic stroke. We report a case of a 74-year-old male patient with uncontrolled diabetes mellitus who presented with right eye pain and proptosis. Imaging revealed a sino-orbital mass with invasion into the cavernous sinus and internal carotid artery. Following a biopsy, the patient suffered an ischemic stroke due to the right internal carotid artery occlusion, requiring mechanical thrombectomy and stent placement. Histopathology confirmed *Aspergillus fumigatus*, and the patient was treated with amphotericin B and voriconazole, showing improvement. This case highlights the importance of early diagnosis and a multidisciplinary approach in managing invasive sino-orbital aspergillosis to prevent fatal vascular complications.

## Introduction

Aspergillus is the most common cause of fungal sinusitis. Noninvasive infections can manifest as sinusitis, leading to bony expansion and the destruction of the sinus mucosa, but without bony invasion [1]. Invasive sino-orbital aspergillosis is an uncommon yet life-threatening disease predominantly seen in immunocompromised patients, including those with diabetes mellitus, long-term steroid use, HIV, or hematological malignancies. The infection often originates in the paranasal sinuses and extends into adjacent structures, leading to orbital and cranial involvement with fatal complications [2,3]. The maxillary and the ethmoid sinuses are the most commonly involved sinuses in aspergillosis. Herein, we present a case of invasive sino-orbital aspergillosis with internal carotid artery (ICA) invasion, resulting in ischemic stroke and requiring urgent endovascular intervention.

## Case presentation

**Figure 1:**
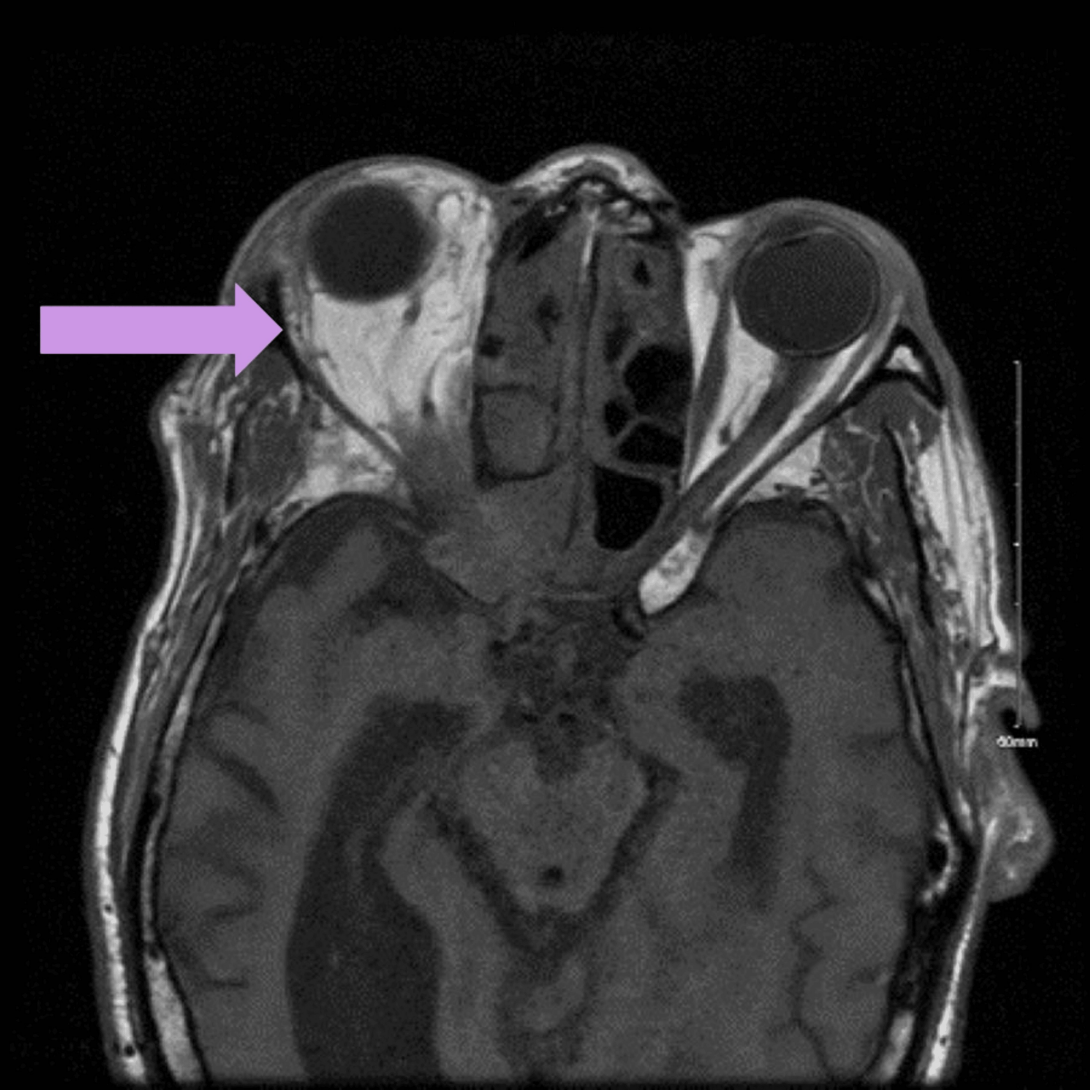
Magnetic resonance imaging of the brain and orbits showing ill-defined infiltrative enhancement centered at the orbital apex involving the right superior, medial, inferior, and lateral rectus muscles, the right orbital floor, anterior clinoid process, cavernous sinus, pterygoid process, masticator space, and temporal fossa.

A 74-year-old male patient with a history of uncontrolled diabetes mellitus presented with generalized weakness, dizziness, right eye pain, and photophobia of one month duration. On physical examination, there was proptosis of the right eye with drooping of the right eyelid. The right eye movements were limited inferolaterally. The visual acuity in the right eye was 16/20 and 20/20 in the left eye. The intraocular pressures were normal in both eyes. There was no facial palsy and no focal neurological deficits. Magnetic resonance imaging (MRI) of the brain and orbits with contrast revealed a sino-orbital mass extending into the optic canal, cavernous sinus, internal carotid artery, and temporal fossa (Figure 1).

**Figure 2:**
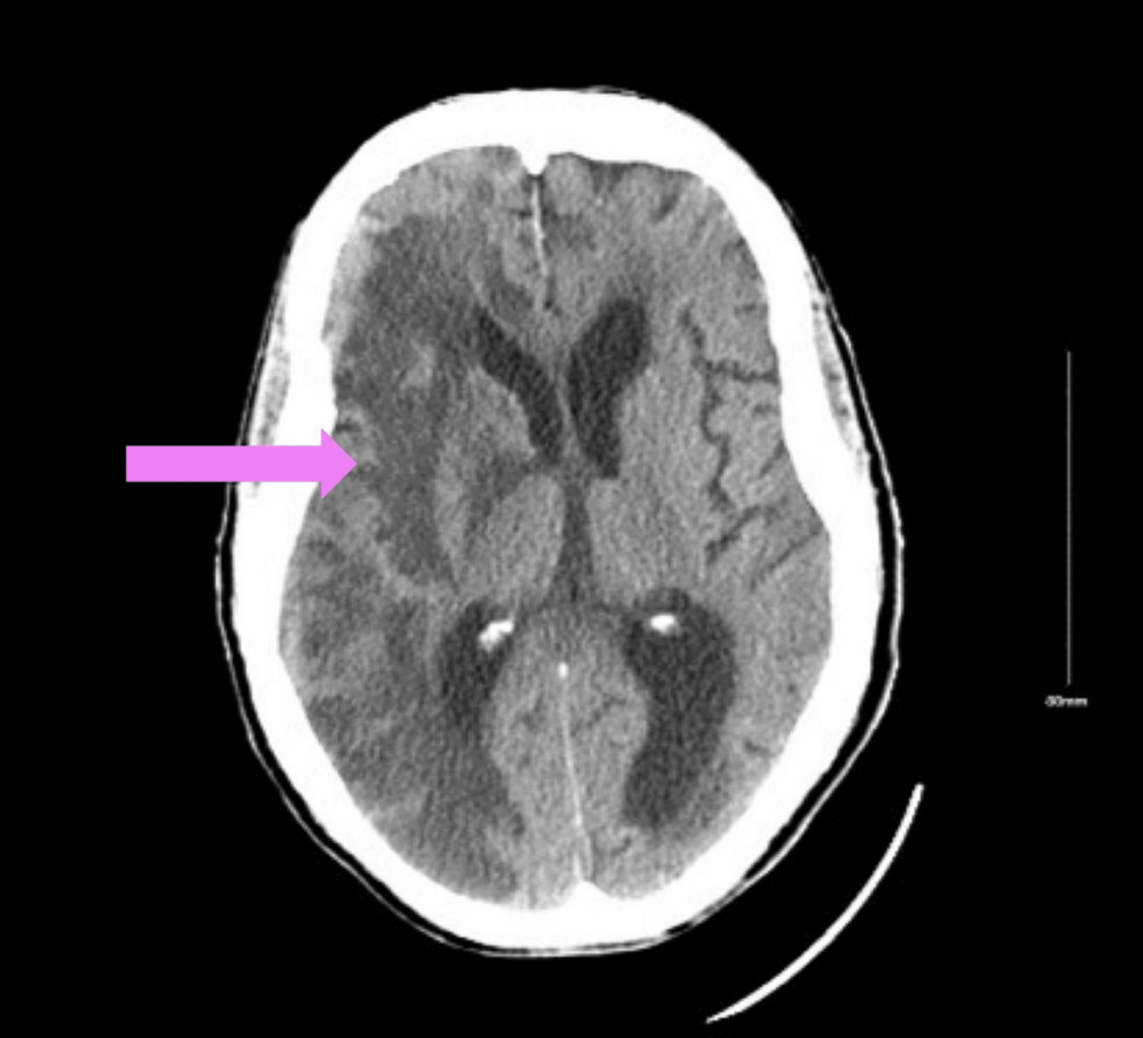
Computed tomography head shows extensive region of infarcted brain parenchyma in the right anterior and middle cerebral artery territories, secondary to right internal carotid artery occlusion.

Blood glucose level was 450 mg/dl. Other laboratory tests, including complete blood count, and liver, and renal functions, were normal. The blood cultures were negative. Given the suspicion of invasive fungal infection on MRI, a multidisciplinary team was involved, including otolaryngology, neurosurgery, ophthalmology, endocrinology, and infectious disease specialists, and the patient was started on amphotericin B. An endoscopic procedure was performed to biopsy the right sphenoethmoidal mass. Shortly after the procedure, the patient developed left-sided hemiparesis with mild sensory loss. The National Institutes of Health (NIH) stroke scale was 16 points. The computed tomography of the head without contrast showed extensive infarct in the right anterior and middle cerebral artery territories (Figure 2). The computed tomography angiogram (CTA) of the brain and neck confirmed right internal carotid artery (RICA) occlusion with thrombus.

The patient underwent an urgent mechanical thrombectomy with RICA stent placement. Histopathological examination of the right sphenoethmoidal mass confirmed invasive *Aspergillus fumigatus* infection. The patient had a progression of disease on amphotericin B. After the confirmation of diagnosis, the patient was initiated on dual antifungal therapy with voriconazole and caspofungin, dual antiplatelet therapy, and high-dose statin. The antifungal treatment was planned for a total of twelve weeks. The patient showed clinical improvement after a few weeks of treatment with resolution of eye pain, diplopia, and dizziness. At the time of the last follow-up, one month after the completion of antifungal therapy, the patient had improved vision in his right eye with partial resolution of proptosis. The left-sided motor deficits were persistent for which the patient was undergoing sub-acute rehabilitation.

## Discussion

Invasive aspergillosis extending from the sinuses to the skull base or cavernous sinus is a fulminant disease that can cause inflammation or invasion of the adjacent ICA, leading to thrombosis, aneurysm formation, or vessel rupture. Invasive fungal carotiditis can lead to ischemic stroke, subarachnoid hemorrhage (SAH), and death [2,4,5].

The most common pathogen implicated is *Aspergillus fumigatus*, a ubiquitous fungus with angioinvasive properties contributing to arterial thrombosis and infarction [6].

Little et al., in their case series of 78 cases of invasive fungal carotiditis, found *Aspergillus *species in 41 cases. The most common presenting symptoms were headache, vision changes, and cranial nerve palsies. Vascular events included occlusion, aneurysm formation, and vessel rupture. Cerebral infarcts occurred in 50% of cases. The mortality was high. Of the patients, 71% died at two years [2].

Baeesa et al. described six immunocompetent patients with invasive orbital apex aspergillosis complicated by subarachnoid hemorrhage (SAH) secondary to ruptured mycotic aneurysms [7]. Two patients had vasospasm and brain infarction. All patients were treated with antifungal therapy, and four underwent emergency craniotomy and clipping of an aneurysm. Despite aggressive management, five patients died of SAH and infarction [7].

Identifying the disease is often complex due to the multitude of possible etiologies and requires orbital and brain imaging and histopathological examination of a biopsy specimen. Isolation of *Aspergillus fumigatus* from pathological tissue is the gold standard for diagnosis. Management typically includes a combination of surgical debridement, systemic antifungal therapy, and endovascular interventions such as thrombectomy or stent placement in cases of vascular involvement [6]. Despite aggressive treatment, the prognosis remains guarded, with high morbidity and mortality reported in advanced cases.

## Conclusions

Invasive Sino-orbital aspergillosis with invasive fungal carotiditis is a life-threatening disease. Prompt recognition is critical, as delays in treatment are associated with high mortality rates. To reduce the high mortality rate, an early aggressive approach to surgical debridement, appropriate antifungal therapy, and endovascular intervention should be considered. This calls for a multidisciplinary approach between neurosurgeons, otolaryngologists, ophthalmologists, and infectious disease specialists.
